# Environmental Evaluation in Bakery and Brewing Sectors in a Circular Economy Context

**DOI:** 10.3390/foods15091611

**Published:** 2026-05-06

**Authors:** Ionică Drăgan, Emilie Korbel, Gaelle Petit, Lynda Aissani, Vanessa Jury

**Affiliations:** 1 Oniris, GEPEA, UMR6144, 44000 Nantes, France; ionica.dragan.1@ulaval.ca (I.D.); emilie.korbel@oniris-nantes.fr (E.K.); vanessa.jury@oniris-nantes.fr (V.J.); 2 IRSTV—Institut de Recherche en Sciences et Techniques de la Ville, FR CNRS 2488, CEDEX 3, 44321 Nantes, France; lynda.aissani@inrae.fr; 3 Rennes Institute of Political Studies, ARENES UMR CNRS 6051, 35700 Rennes, France; 4 French National Institute for Agriculture, Food, and Environment (INRAE), UR OPAALE, 35044 Rennes, France

**Keywords:** bread, beer, life cycle assessment, life cycle thinking, upcycling, sustainable agri-food chains

## Abstract

Ensuring sustainable food production for a growing population requires robust tools like the Life Cycle Assessment (LCA), despite the fundamental complexities characterising the agri-food sector. This study evaluates the environmental impacts of beer and bread production, two important sectors, within a circular economy framework using the LCA. The analysis focuses on innovative products: bread incorporating brewery-spent grain and beer brewed from unsold bread. The study follows a cradle-to-gate approach, covering the entire upstream supply chain, including cultivation, milling, malting, and ingredient production. Cultivation emerges as the primary environmental hotspot in both systems. In bread production, the bakery and proofing phases also show high impacts, while in brewing, packaging is the dominant contributor, followed by boiling and hopping. For co-product processing, drying and transport are critical hotspots. Compared with conventional products, innovative circular products generally show lower environmental impacts, with exceptions related to organic cultivation and allocation constraints. Circular strategies notably reduce land use and marine eutrophication in most organic cases. Overall, the fully circular scenario outperforms the Conventional System in 13 impact categories, supporting the environmental potential of circular approaches in both sectors.

## 1. Introduction

The agri-food sector holds significant economic, social, and environmental importance. However, it must address the critical challenge of feeding an increasing world population with food that is safe, healthy, and nutritious, while simultaneously limiting impacts on ecosystems, natural resources and human health [1]. In this context, the brewing and baking sectors hold a particularly significant position both within the European Union and worldwide [2,3,4].

Beer is the most commonly consumed alcoholic drink worldwide and the two most popular cultivated grains used are barley and wheat, which are typically processed through the malting process [5]. The other essential ingredients for brewing are water, hops, and yeast. Furthermore, beer production generates around 8 million tonnes of brewer’s spent grain (BSG) each year in Europe and 40 million tonnes worldwide. Recovering this material represents a valuable opportunity within the shift toward sustainable food systems, as BSG is rich in proteins, fibres, and other nutrients relevant for human nutrition [6].

On the other hand, the baking sector is one of the food industries experiencing the most rapid growth and bread stands out as a staple food, valued for its rich flavour, nutritional content, and essential role in the human diet [7]. However, traditional bread, primarily made with white wheat flour, lacks essential nutrients. This has driven innovations to improve its nutritional value by adding fibres, proteins, vitamins, and minerals [7]. Bread production is estimated at roughly 100 million tonnes annually, of which 65% is consumed in Europe [3]. Worldwide, food waste results in important economic losses, and bread ranks among the most wasted food items in developed nations, with nearly 10% of the annual production being discarded [8,9,10].

Despite the well-documented potential of BSG in bakery applications, particularly in bread products [11,12,13], and the reuse of surplus bread in brewing as a strategy to reduce food waste [3,14], comprehensive environmental impact assessments integrating these two systems remain scarce. Available studies mainly focus on the following aspects:

- The potential use of co-products in food formulation [3,6,11,13,15,16,17];
- LCA of singular conventional products, beer and bread [5,18,19,20,21,22,23,24,25,26];
- LCA of a singular innovative product without the circular context: beer bread [14].

This study aims to address that unexplored area by assessing the environmental implications of combining bread and beer production within a circular production model. Considering the environmental burden of the food system [1], the study supports mitigation strategies by identifying process hotspots, comparing products, and promoting valorisation and waste reduction through the Life Cycle Assessment (LCA), a well-established method that produces credible, science-based information for decision processes [1,27,28]. For the above reasons, the study compares innovative circular scenarios with conventional, linear production models. This study makes three novel contributions. First, it provides a joint environmental assessment of two complementary circular strategies (BSG valorisation in bread-making and unsold bread reuse in brewing) within a single integrated LCA framework, while prior work has examined these flows in isolation. Second, it systematically compares organic and conventional production pathways within this circular framework. Third, it investigates the influence of different allocation approaches on the environmental outcomes of these circular systems, thereby contributing methodological transparency to a domain where allocation choices are known to influence conclusions [27,28].

## 2. Materials and Methods

### 2.1. System Description: Products and Processes

#### 2.1.1. Products

**Figure 1:**
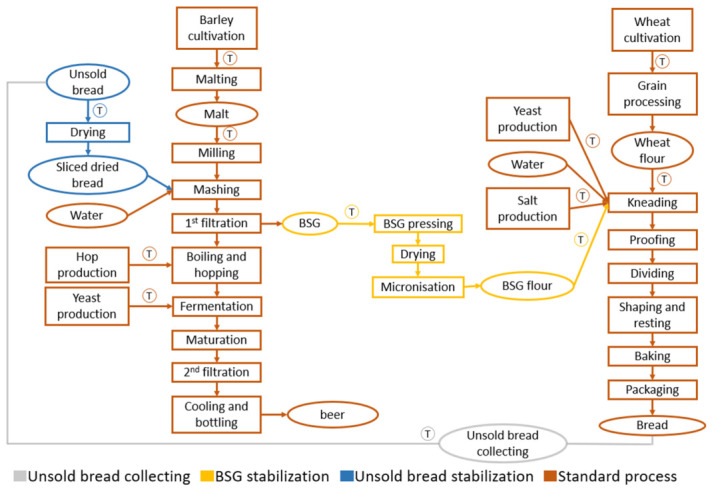
The production system shows three components: traditional processes, unsold bread stabilisation, and brewery-spent grain (BSG) stabilisation, with transport indicated as T.

The study context includes pilot scales and companies in the north-west and south-west of France. For this study, 10 products were considered. A total of 2 traditional breads: traditional baguette (TB) and traditional baguette organic (TB org); 2 traditional beers: beer 100% barley malt (beer) and beer 100% barley malt organic (beer org); 2 upcycled breads: BSG bread—20% of BSG (BSG) and BSG bread organic—20% of BSG (BSG org). Finally, 4 upcycled beers: beer bread—35% barley malt replacement (b.b. 35 ab), beer bread—35% barley malt organic replacement (b.b. 35 ab org), beer bread—50% barley malt replacement (b.b. 50 ab) and beer bread organic—50% barley malt replacement (b.b. 50 ab). Figure 1 shows the flow chart that represents all the products concerning beer production, bread production and the new processes highlighting the various types of processes with different colours. The following paragraphs present the parameters of the evaluated processes.

#### 2.1.2. Beer Production

Beer operations included in the study are reported in Figure 1, brown colour—left. The consumption phase, cleaning products, and bottle recycling were excluded, while hop and yeast production were included. Process details are outlined below.

Cultivation: the farm–malt house transport distance was assumed to be 100 km for modelling purposes. This distance is consistent with typical regional supply chains in France, where barley fields and malting facilities are generally located within similar ranges.

Malting: about 100 kg of barley produces 75 kg of malt through soaking (green malt) and kilning (30 h at 45 °C and later 5 h at a higher temperature), followed by degemination and 2–3 weeks of ageing.

Brewery operations: this study focuses on ale beer, produced by crushing malt and mixing it with water to obtain wort, heated 50–80 min at 64–69 °C. The wort was filtered, boiled 1–2 h, then fermented with *Saccharomyces cerevisiae* at 18–25 °C. After fermentation, the beer was cooled to 5 °C for maturation, filtered again, and bottled. Yeast converts available sugars to alcohol and CO_2_ during fermentation. Inputs/outputs details are in Table A1 in Appendix A.

#### 2.1.3. Bread Production

Bakery operations considered in this study are reported in Figure 1, brown colour—right. Agricultural machinery production was included; company facilities were excluded, except for the milling hall in the global milling process. Milling varies by flour type (55 and 150, white and wholemeal). Bran and germ used for animal feed and cleaning products were excluded, with mass allocation applied. Process details follow.

Soft wheat cultivation and raw ingredients: the ingredients were yeast (Saccharomyces cerevisiae), wheat and salt.

Grain milling: Pre-cleaning was performed. About 75% of grain became white flour, while wholemeal flour retained nearly 100% of the grain.

Bread-making: the approach was guided by the specifications for the production of the traditional baguette [29]. Kneading lasted 8 min at 25 °C (50 tr/min), followed by 1.5 h of proofing. After division, the loaf rested for 15 min at room temperature, then was shaped, rested for 30–45 min at 27 °C, and baked at 240 °C for 20 min. Production data are in Table A2 in Appendix A.

#### 2.1.4. New Processes to Link Both Productions

In order to link the bakery process with the brewery one, the unsold bread collected from supermarkets and bakeries was dried to below 10% of the moisture content. After the brewery process, it was necessary to stabilise the BSG by pressing, drying, micronization and packaging. For these operations, the capacity per batch was assumed to be 300 kg and the duration almost 8 h.

### 2.2. Life Cycle Assessment

#### 2.2.1. Goal and Scope

The LCA was conducted in accordance with ISO 14040 and ISO 14044 (2006) [27,28]. The purpose of this study is to determine which product variants generate the highest environmental impacts, to identify the key process hotspots, and to evaluate whether circular strategies outperform conventional production models. Functional units were defined in line with the literature as 1 kg of bread for bakery scenarios and 1 L of beer for brewery scenarios [5,22], both assessed using a cradle-to-gate perspective. It includes the production and distribution of raw materials, packaging, energy, water and transport along the entire supply chain per unit of co-product stabilisation, baking and brewing operations. Data consistency was ensured through a process-based LCA methodology, which generated detailed inventories for each individual life cycle stage. The following part outlines the assumptions adopted in this study.

- The study considers the brewery and the BSG stabilisation operations to take place in the same locality.
- Elementary flows and processes sources, including electricity, heat, water supply, and transportation, were considered identical across all scenarios.
- No environmental burden was attributed to BSG or unsold bread in the product impact calculations. This approach is common in the LCA when such co-products are treated as waste streams with no remaining economic value avoiding double-counting of cultivation and production impacts [27,28].
- In the organic scenarios, the only parameter that differed from the Conventional System was the cultivation of grains; both yeast and hops continued to originate from conventional production.
- No variability in BSG composition across different processes was assumed. It was necessary because detailed compositional data for BSG originating from each individual brewing process were not available and treating BSG as compositionally uniform ensures methodological consistency within the life cycle model.
- The Waste Me Up bags were excluded from the assessment due to the lack of suitable data.
- The drying operation for unsold bread and BSG relied on the same equipment.
- The 1:1.25 conversion ratio applied to unsold bread serves only to determine the amount of barley avoided, following starch-based equivalence data according to Brussels Beer Project company consultation.
- In the organic scenarios, wheat and barley were modelled as intercropping systems according to AGRIBALYSE v3.0.1: organic wheat cultivated after alfalfa, and organic barley after an unspecified preceding crop.
- The formulation adopts a 1:1 ratio between BSG and wheat flour [30].
- The transport distance for wheat flour, salt and yeast was set to 30 km because no product-specific transport data were available.
- The only modified parameter was the adoption of organic wheat, while distinguishing between wholemeal and white flour remained essential for an accurate evaluation of the milling process.

Finally, a logical framework was developed to enable systematic comparison between three alternative system configurations. Table 1 provides a concise methodological overview of the Conventional System (CS) and the two circular scenarios, the Environmental Charged Circular System (ECCS) and the Without Environmental Charging Circular System (WECCS).

The overview summarises the modelling choices, including system boundaries, the nature of the link between brewery and bakery processes, the treatment of co-products (brewery-spent grain and unsold bread), and the corresponding allocation approaches. This synthesis is intended to clarify the conceptual differences between scenarios and to support the interpretation of results presented in the following sections.

#### 2.2.2. Life Cycle Inventory

The LCA was conducted using OpenLCA v1.11.0. Primary data were collected for all production stages, including malting, brewing, BSG stabilisation, bread stabilisation, and bakery operations. Secondary datasets were sourced from ECOINVENT v3.9.1 and AGRIBALYSE v3.0.1 (as part of the AGRIBALYSE programme lead by ADEME and INRAE since 2009, France). Data related to cultivation, transport, and grain milling in bread production were obtained from these databases, incorporating the adjustments proposed by Montemayor et al. and Heshe et al. [31,32]. Transportation of ingredients was modelled using the Ecoinvent process “*Transport*,* freight*,* lorry 3.5–7.5 metric ton*,* EURO5 {GLO} | market for | Cut-off*,* S*” while transportation of BSG followed the process “*Market for transport*,* freight*,* and light commercial vehicle*”. Unsold bread stabilisation activities were modelled based on Morgan et al. [5]. Ingredient transport distances were calculated excluding empty return trips. According to the literature [3,11,13], the proportion of unsold dried bread used in new beer formulations is 35% and 50%, while BSG bread incorporates 20% of BSG. The packaging data (bottle and kraft paper), hop pellets and yeast production were from ECOINVENT v3.9.1. OpenLCA’s empty processes and programmes were populated using SimaPro 9.3 datasets to ensure completeness and consistency. The detailed Life Cycle Inventories for 1 kg of BSG-20% and 1 L of b.b. 35 and 50 ab are provided in Table A1 and Table A2 of Appendix A.

#### 2.2.3. Environmental Impact Method

The Life Cycle Inventory Analysis (LCIA) method used was ReCiPe 2016 Midpoint (H). Therefore, 18 environmental impact categories (EICs) were considered: Fine particulate matter formation (FPMF), Fossil resource scarcity (FRS), Freshwater ecotoxicity (FE), Freshwater eutrophication (Feutr), Global warming (GW), Human carcinogenic toxicity (HTC), Human non-carcinogenic toxicity (HNCT), Ionising radiation (IR), Land use (LU), Marine ecotoxicity (ME), Marine eutrophication (Meutr), Mineral resource scarcity (MRS), Ozone formation, Human health (OF,HH), Ozone formation, Terrestrial ecosystems (OF,TE), Stratospheric ozone depletion (SOD), Terrestrial acidification (TA), Terrestrial ecotoxicity (TE), and Water consumption (WC). Considering all assumptions, a threshold of 20% was established in accordance with guidance from the cited sources [33,34] (Table A3 in Appendix A for more details regarding the units used).

## 3. Results and Discussion

### 3.1. Brewery

#### 3.1.1. Hotspots

**Figure 2:**
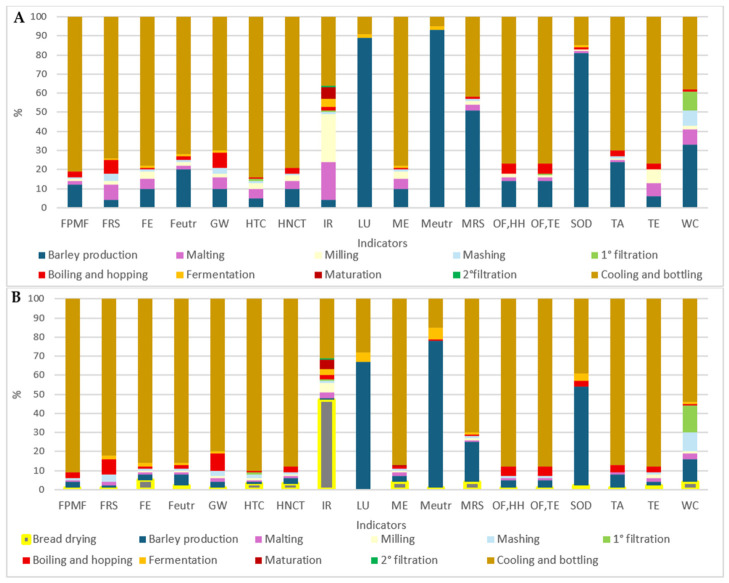
Stacked bar chart of percentage contribution of beer 100% barley production process (**A**) and of b.b. 35% (beer bread avoided barley 35%) (**B**) for each environmental impact category. FPMF: fine particulate matter formation, FRS: Fossil resource scarcity, FE: Freshwater ecotoxicity, Feutr: Freshwater eutrophication, GW: Global warming, HTC: Human carcinogenic toxicity, HNCT: Human non-carcinogenic toxicity, IR: Ionising radiation, LU: Land use, ME: Marine ecotoxicity, Meutr: Marine eutrophication, MRS: Mineral resource scarcity, OF,HH: Ozone formation, Human health, OF,TE: Ozone formation, Terrestrial ecosystems, SOD: Stratospheric ozone depletion, TA: Terrestrial acidification, TE: Terrestrial ecotoxicity, WC: Water consumption.

All percentage contributions of EICs of a conventional production are presented in Figure 2A. The packaging stage (cooling and bottling) emerges as the primary hotspot, dominating nearly all EICs with more than 70% contribution in 12 out of 18 EICs. This is largely due to the absence of glass recycling in the study, forcing the model to treat all glass as virgin material, which has high environmental impacts, as highlighted by Heller et al. because of “not avoided burden”. The mentioned study establishes a hierarchy of packaging types in breweries and identifies non-recycled glass bottles as the most environmentally burdensome option [35]. Similarly, Cordella et al. also found glass bottling to be the most impactful stage in a the LCA [19]. Following packaging, barley cultivation is a major contributor to several EICs, accounting for 89% of LU, 81% of SOD, 93% of Meutr, and 51% of MRS.

The findings align with those reported by Morgan et al., who also highlight the cultivation phase as a significant environmental hotspot in the beer life cycle in acidification, TE, and Meutr [5]. The malting and milling steps also represent critical points, especially for IR, with respective contributions of 20% and 25%, largely due to intensive energy—0.14 kWh and 0.04 kWh respectively—and resource consumption—2.41 MJ (for malting). According to Cimini and Morensi, in large and medium-sized breweries, the manufacturing and transportation of glass bottles represents the primary environmental burden, followed closely by barley cultivation and its conversion into malt. In contrast, for small-sized breweries, the main hotspots shift, aligning instead with those associated with beer packaging in polyethylene terephthalate (PET) or glass bottles [36]. Within the brewing process, the boiling phase is particularly energy-intensive (0.40 MJ) and contributes notably to both TE and IR.

Notably, if packaging was excluded or recycled, the relative contributions of the aforementioned hotspots would considerably increase, as reported by Morgan et al. [5]. However, Cordella et al. report that the brewing stage does not constitute a critical hotspot in the overall beer life cycle [19]. For the innovative product, a “bread beer” made with 35% bread at the mashing step, the impacts and corresponding hotspots are shown in Figure 2B. In terms of relative contribution, it remains similar to the conventional ale beer except for “boiling and hopping” that increase its contribution (9% in GW and 8% in FRS). However, in absolute values, this formulation reduces the use of barley malt by approximately 35%, thereby lowering the contributions from barley cultivation, malting and milling. When packaging is excluded, the impact contribution obviously changes: barley cultivation becomes the main contributor, especially for LU, Meutr, and SOD. Additionally, “boiling and hopping” and “drying bread” emerge as important hotspots. In fact, according to Brancoli et al. and Almeida et al., the main hotspots remain residual barley production, bread transportation (it might be far from the microbreweries), energy consumption (particularly when sourced from non-renewable sources) and co-product management (specifically avoiding landfill disposal) [14,17]. While using surplus bread reduces the impact of barley, energy efficiency and effective waste recovery remain important to minimising the overall environmental footprint.

#### 3.1.2. Beer Products Comparison

**Figure 3:**
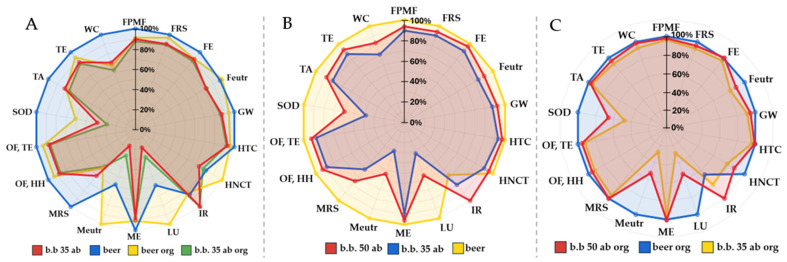
Radar chart comparing the environmental impact categories of: conventional beer, b.b. 35% ab (beer bread 35% avoided barley) and respective organic production (**A**); conventional beer and b.b. 35% and 50% ab (beer bread 35% and 50% avoided barley) (**B**) and the environmental impact categories of organic beer and b.b. 35% and 50% ab (**C**). Every figure is independent and must be analysed separately. For each environmental impact category, the value corresponding to 100% represents the highest value within that specific category and the rest is relative to it. FPMF: fine particulate matter formation, FRS: Fossil resource scarcity, FE: Freshwater ecotoxicity, Feutr: Freshwater eutrophication, GW: Global warming, HTC: Human carcinogenic toxicity, HNCT: Human non-carcinogenic toxicity, IR: Ionising radiation, LU: Land use, ME: Marine eco-toxicity, Meutr: Marine eutrophication, MRS: Mineral resource scarcity, OF,HH: Ozone formation, Human health, OF,TE: Ozone formation, Terrestrial ecosystems, SOD: Stratospheric ozone depletion, TA: Terrestrial acidification, TE: Terrestrial ecotoxicity, WC: Water consumption.

These findings highlight the trade-offs introduced by the use of surplus bread, emphasising both its benefits and its limitations across different impact categories. To better understand the overall performance of bread-based beers, Figure 3A compares the environmental impacts of various beer products including innovative, organic, and conventional formulations. Among all evaluated beer formulations, the least impactful are b.b. 35 ab org and b.b. 35 ab. Their GW potentials range from 663 g CO_2_ eq./L for b.b. 35 ab org to 771 g CO_2_ eq./L for conventional beer. For the FRS, it ranges from 196 g oil eq./L to 220 g oil eq./L. Comparing b.b. 35 ab with conventional beer, LU is reduced by 40%, WC by 29%, Meutr by 66% and MRS by 33%. Concerning organic productions (beer org and b.b. 35% org), the benefits of adding unsold bread during mashing are especially prominent in LU, in Meutr and MRS; categories usually associated with a higher impact for organic production [37,38].

A preliminary assessment was conducted by comparing conventional beer with formulations incorporating progressively higher proportions of dehydrated bread, as illustrated in Figure 3B (Table A4 in Appendix A for more information). The results show that increasing the proportion of dehydrated bread does not produce a linear or proportional decrease in EICs. While conventional beer remains the most impactful formulation, the lowest impacts are observed for the b.b. 35 ab variant rather than the b.b. 50 ab formulation. It can be explained by the combined effects of avoided burdens, additional processing requirements, and energy-driven trade-offs. Energy demand associated with bread stabilisation (drying and transport) increases almost linearly with the amount of unsold bread incorporated. Drying alone represents a significant energy input. As a result, in the 50% substitution scenario, the additional energy required for drying outweighs the marginal environmental benefit obtained from further reducing malt use, leading to higher total impacts in several categories compared with the 35% formulation. Higher substitution rates also imply larger quantities of unsold bread to be collected and transported.

A direct comparison between conventional beer and the b.b. 35 ab formulation shows substantial reductions across several EICs: LU decreases by 68%, Meutr by 70%, MRS by 40%, SOD by 62%, and WC by 29%. These improvements are partly offset by the additional energy demand associated with drying (0.64 kWh) and by the transport of unsold bread (10,000 kg × km). Nonetheless, the overall reductions are primarily driven by the avoided malt production (0.076 kg), which significantly lowers the environmental burden of the b.b. 35 ab product.

The comparison between the two innovative products (Figure 3C), b.b. 35 ab and b.b. 35 ab org, reveals that the performance gap typically observed between conventional and organic formulations diminishes when considering these new products. When comparing traditional and innovative options, the most affected EICs include HNCT (22% reduction for the organic product), LU (71% reduction), Meutr (72% reduction), and SOD (53% reduction) relative to conventional beer. These improvements result largely from the avoided product burdens; however, not all EICs exhibit reductions, as certain categories increase due to the substantial energy demand associated with the drying process (4.17 kWh). In organic traditional beer, the LU and the Meutr are notably high because of cultivation yield. The partial replacement of barley malt with surplus bread can be considered environmentally beneficial, primarily due to the reduction in the carbon footprint and the repurposing of food waste. When bread is not consumed, all the resources used to produce it have been wasted and it does not generate new emissions due to waste management [3,17]. It is important to highlight that the impacts related to wheat cultivation and bread-making (the “upstream” stages of bread production) were not included in the beer calculation. The “burden-free” approach is justified by treating surplus bread as a genuine waste stream with no remaining economic value, whose only alternative fate would be landfill disposal. Because the bread has already completed its primary life cycle, no upstream impacts from wheat production or baking are allocated to it. Instead, only the additional environmental burdens linked to its recovery and reuse, such as energy required for drying before mashing, are included, meaning that the beer accounts solely for impacts directly caused by the brewing process and the waste pre-treatment.

Almeida et al. report that beer brewed with surplus bread achieves a 20% lower carbon footprint compared to standard craft beer. While their study acknowledges that replacing malt with waste bread supports sustainability, it emphasises that the greatest environmental benefits stem from circular economy practices, particularly the reuse of BSG as livestock feed instead of landfill disposal [14]. By contrast, the present study focuses on a different scenario, prioritising human nutrition rather than animal feeding.

The last comparison is between organic products reported in Figure 3C (Table A4, Appendix A). Increasing the proportion of unsold bread does not translate into a proportional decline in EICs. Instead, the organic beer brewed with 35% dried bread stands out as the most efficient option, delivering reductions exceeding 20% in IR, LU, Meutr, and SOD compared with other organic formulations.

### 3.2. Bakery

#### 3.2.1. Hotspots

**Figure 4:**
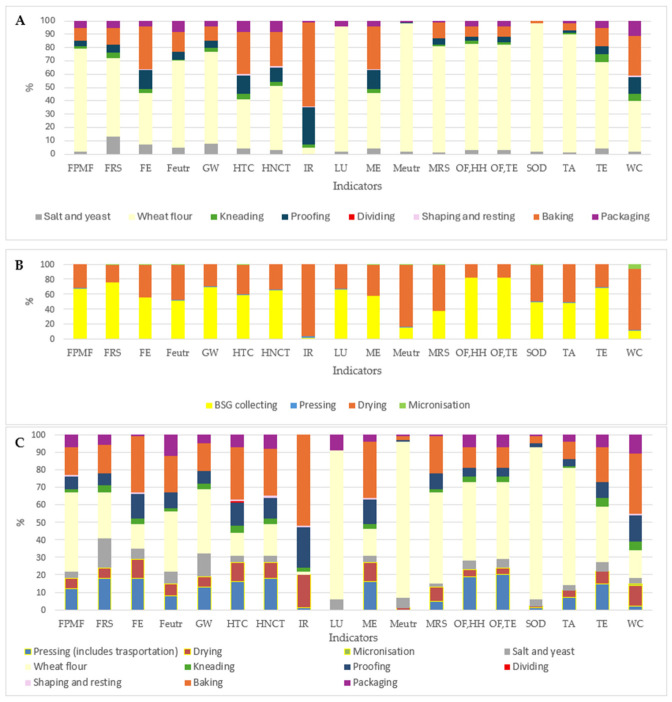
Stacked bar chart of percentage contribution of traditional baguette process (**A**), of BSG (brewery-spent grain) stabilisation process (**B**), and of BSG bread production process (**C**) for each environmental impact category. FPMF: fine particulate matter formation, FRS: Fossil resource scarcity, FE: Freshwater ecotoxicity, Feutr: Freshwater eutrophication, GW: Global warming, HTC: Human carcinogenic toxicity, HNCT: Human non-carcinogenic toxicity, IR: Ionising radiation, LU: Land use, ME: Marine eco-toxicity, Meutr: Marine eutrophication, MRS: Mineral resource scarcity, OF,HH: Ozone formation, Human health, OF,TE: Ozone formation, Terrestrial ecosystems, SOD: Stratospheric ozone depletion, TA: Terrestrial acidification, TE: Terrestrial ecotoxicity, WC: Water consumption.

In conventional bread production, wheat flour is by far the dominant contributor to environmental impacts, encompassing cultivation, milling, and grain storage, and it drives the majority of EICs, as illustrated in Figure 4A (e.g., 69% GW; 39% Feutr; 80% MRS; 80% OF,HH and 89% TA). Similar findings were reported in the explored literature, and in many cases it represents the 50% of the total impact in different categories [26,39].

The main environmental impacts of wheat cultivation come from nitrogen fertilisation, which causes emissions and water pollution, the energy-intensive production of fertilisers, and the use of diesel in field operations. It is followed by baking (32% FE; 32% HTC; 63% IR; 32% ME and 30% WC) and proofing (FE 14%; HTC 14%; IR 28%; ME 14% and WC 13%), which are energy-intensive processes, while the other processes, salt and yeast production, contribute minimally to the overall impact since these steps run for only a brief period and thus impose an extremely low energy demand. It is possible to find similar conclusion in the literature [22,25,26,39].

Regarding BSG stabilisation (shown in detail in Figure 4B), the most impactful stage across all EICs is the collection of BSG, primarily due to inefficient transportation, as noted by Morgan et al. [5]. This is followed by the drying process, which requires substantial energy, largely from nuclear sources, with additional environmental pressure linked to the cooling demands of nuclear reactors. As Petit et al. suggested, scaling and improving the energy efficiency of the drying technology could reduce these impacts [6].

An environmental assessment was also carried out for the innovative 20% BSG bread. As shown in Figure 4C, the principal hotspots of this product remain largely driven by wheat-flour-related processes, particularly cultivation (37% GW; 89% Meutr; 45% OF,HH; 32% TE) and the baking step (32% FE; 30% HTC; 21% Feutr; 52% IR; 32% ME; 34% WC). Bakery operations are generally less impactful than the drying step and BSG collecting has a notable impact on “BSG stabilisation” and on overall production. Although BSG stabilisation is not the dominant contributor to any single EIC, this additional processing stage still exerts a substantial overall influence on the product’s environmental profile (more than 20% for the majority of EICs), again, in accordance with Petit et al. [6].

#### 3.2.2. Bread Products Comparison

**Figure 5:**
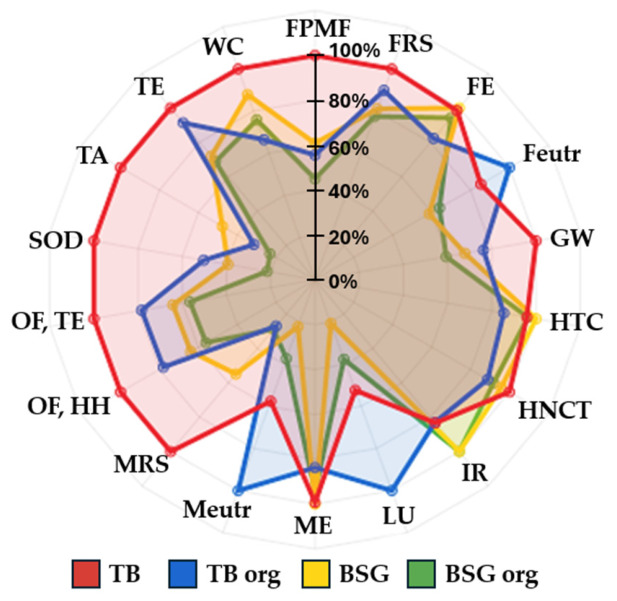
Radar chart comparing the environmental impact categories of: all bread products comparison. TB: Traditional bread, TB org: traditional bread organic, BSG: Brewery-spent grain bread, BSG org: Brewery-spent grain bread organic. FPMF: fine particulate matter formation, FRS: Fossil resource scarcity, FE: Freshwater ecotoxicity, Feutr: Freshwater eutrophication, GW: Global warming, HTC: Human carcinogenic toxicity, HNCT: Human non-carcinogenic toxicity, IR: Ionising radiation, LU: Land use, ME: Marine eco-toxicity, Meutr: Marine eutrophication, MRS: Mineral resource scarcity, OF,HH: Ozone formation, Human health, OF,TE: Ozone formation, Terrestrial ecosystems, SOD: Stratospheric ozone depletion, TA: Terrestrial acidification, TE: Terrestrial ecotoxicity, WC: Water consumption.

Figure 5 presents a comparison of four bread products (TB, TB org, BSG bread, and BSG bread org). Among these, TB shows the highest impact across almost all EICs, whereas BSG bread exhibits the lowest, with minimal differences observed between its conventional and organic versions. This reduction is primarily due to the avoided cultivation of wheat. For instance, in terms of GW, values range from 560 g CO_2_ eq. for TB to 300 g CO_2_ eq. for BSG bread org. Similar substantial reductions are observed in FPMF, MRS, SOD, and TA. In relative terms, BSG and BSG org are the least impactful options, mainly due to the elimination of flour production. The most notable reductions (BSG vs. TB) include: FPMF by 46%, FRS by 21%, Feutr by 37%, GW by 37%, LU by 69%, Meutr by 70%, MRS by 52%, OF,HH by 43%, OF,TE by 42%, SOD by 69%, TA by 60%, and TE by 30%. These reductions occur despite the high energy demand of BSG stabilisation which is 4.3 kWh (pressing, drying, micronization). Although comprehensive environmental comparisons between conventional bread and BSG-integrated bread are scarce (or often limited to comparisons of BSG versus conventional ingredients without full process evaluation), the observed trends align with findings reported in the literature [40,41].

The TB org product follows typical patterns seen in organic crops: higher values in Feutr, Meutr, and LU compared to TB, but lower values in other EICs. Conversely, the BSG bread org product shows considerable reductions, benefiting from the avoided production of raw materials. For example, Meutr decreases from 2 × 10^−3^ kg N eq. (TB org) to 9 × 10^−4^ kg N eq. (BSG bread org), while LU and Feutr also show considerable reductions, typically problematic for organic productions [42,43]. When compared to the control (TB org), BSG bread org shows a clear environmental benefit, with reductions of: FE by 36%, GW by 22%, LU by 62%, Meutr by 63%, OF,HH by 28%, OF,TE by 28%, SOD by 57%, TA by 26%, and TE by 25%.

The difference between BSG org and BSG is below 20% except for TA; MRS and SOD. Finally, comparing TB org to TB highlights considerable improvements in TA (−64%), SOD (−49%), and MRS (−71%), but also increased impacts in Meutr, LU, and Feutr, which are common challenges in organic agriculture. All the numerical values of the impacts are reported in Table A5, Appendix A.

### 3.3. System Comparison

The last step is to compare the entire innovative system with its non-innovative counterpart, thereby assessing the feasibility of circular economy integration from an environmental standpoint. The BSG bread 20% and b.b. 35% ab variants were selected because they constitute the most representative options and are the easiest for manufacturers to reproduce. Particularly, the b.b. 35% ab formulation is the environmentally most efficient compromise among the tested beer-bread scenarios. Moreover, from an industrial perspective, the 35% substitution level represents a technically robust and more readily scalable option for breweries, whereas the 50% formulation may face constraints related to process stability (from the consultation with the partner Brussels Beer Project).

**Figure 6:**
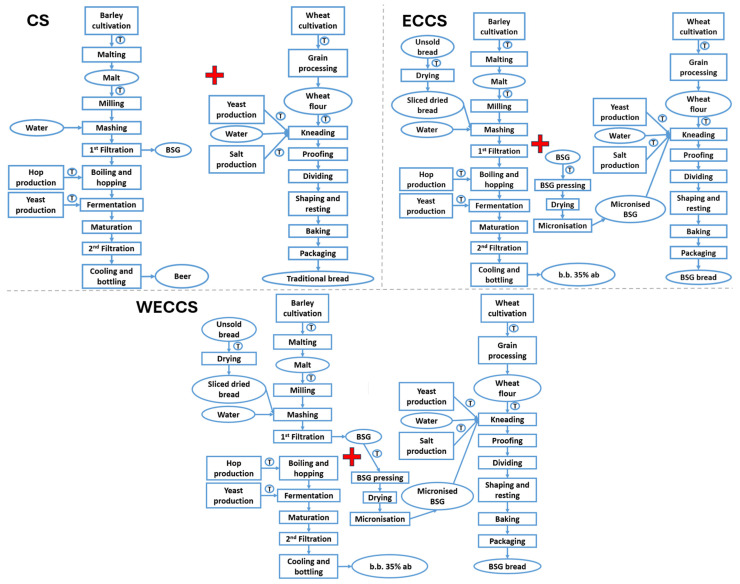
System comparison, flowchart illustration: non-circular system (Conventional System—CS); circular system (with environmental charging of brewery-spent grain: Environmental Charged Circular System–ECCS); circular system (without environmental charging: Without Environmental Charging Circular System—WECCS).

A major limitation in LCA modelling concerns the ability to derive the EIC values for the entire circular economy system and to compare them with the corresponding EIC values of the fully non-circular system, which is composed of two distinct production processes. To address this challenge, the adopted approach consists of summing the EIC values of the bakery process with those of the brewery process. Two summation strategies are implemented and subsequently compared with the CS: (i) integrating the two processes into a single production chain by linking the first filtration stage to the BSG pressing step and then adding the remaining brewing operations (ECCS); and (ii) summing the two productions independently, without assigning any environmental burden to co-products (WECCS). For clarity, the corresponding flowcharts are provided in Figure 6.

In the life cycle inventory, each unit process is modelled so that it generates a defined intermediate output corresponding to the reference flow of that process. When moving to a subsequent process, only the required quantity of this intermediate output is withdrawn and used as input according to the formulated production. As a result, both material and energy balances are inherently preserved within the process-based LCA framework, as all inputs and outputs remain quantitatively proportional to the intermediate flows exchanged between processes. Consequently, the associated environmental impacts are also automatically scaled in direct proportion to these material and energy flows throughout the system.

The comparison conducted in this study is based on mass, as both functional units (1 kg of bread and 1 L of beer) are approximately equivalent in mass. Indeed, considering the density of beer, which is close to 1 kg × L^−1^, 1 L of beer corresponds approximately to 1 kg. The adoption of different functional units reflecting nutritional equivalence (e.g., per kcal or per nutritional value) was not considered, as it falls outside the scope of this study. The primary function of bread is nutritional sustenance, whereas beer primarily fulfils a hedonic or consumption-driven role rather than a nutritional one. Consequently, comparing the two products on a nutritional basis would not be functionally meaningful.

For these reasons, a mass-based comparison represents the most appropriate and neutral basis for the system-level analysis performed in this work. Table 2 presents the EIC values for all systems, along with their corresponding percentages, calculated using the highest EIC value as the 100% reference point. Consequently, the lowest percentage value is relative to the highest value (which is expressed as 100%) for each EIC. CS serves as the reference method for pairwise percentages comparison with ECCS and WECCS respectively.

**Figure A1:**
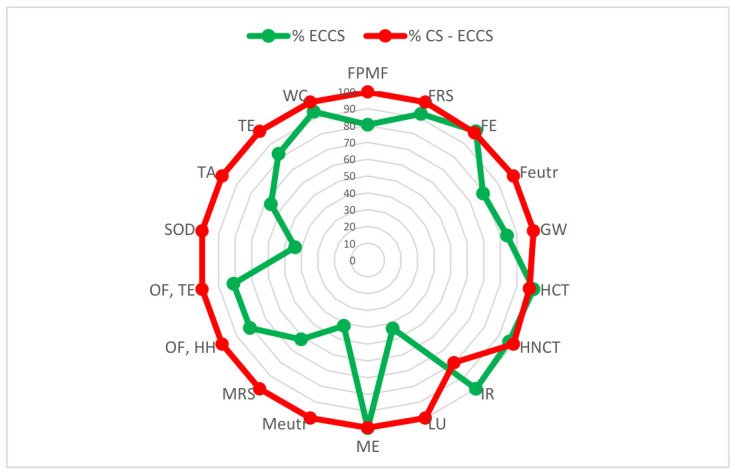
First strategy—with environmental charging circular system (ECCS—green) in comparison with the Conventional System (CS—red).

**Figure A2:**
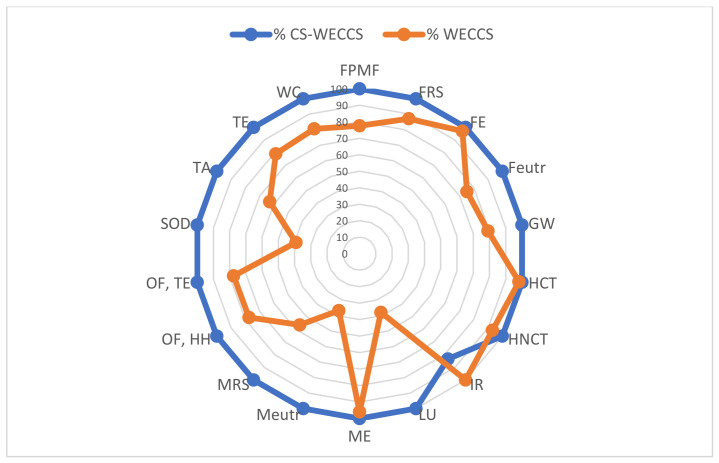
Second strategy—without environmental charging comparison system (WECCS—orange in comparison with the Conventional System (CS—red).

The values highlighted in green represent the most influential results, defined as reductions exceeding 20% relative to the reference value of 100%, which corresponds to the Conventional System (CS), with the exception of IR. Under the first strategy (ECCS), the most significant improvements are observed for FE, LU, Meutr, MRS, SOD, and TA. IR, highlighted in red, is the only EIC that exceeds the impact of the CS by more than 20%. Although WECCS and ECCS show no substantial divergences, the second strategy (WECCS) encompasses additional EICs exhibiting notable reductions, including FPMF, GW, OF,HH, OF,TE, and TE. Importantly, this strategy does not generate any major adverse environmental effects. For a more comprehensive interpretation of the differences between the two circular strategies and the CS, the radar charts presented in Figure A1 and Figure A2 of Appendix B should be consulted.

## 4. Conclusions

This study demonstrates how circular economy strategies can be effectively applied within the brewing and baking sectors by redirecting under-utilised by-products (BSG and unsold bread) back into food production. By integrating these valorisation pathways within an LCA framework, the analysis highlights the environmental relevance of coupling waste reduction with resource substitution in agri-food chains in a cradle-to-gate approach. Beyond the specific case studies assessed, several general principles of circular economy in the food industry emerge from the results. First, avoiding primary agricultural production through the reuse of existing by-products is a decisive lever for reducing impacts, particularly for LU, Meutr, and MRS. Second, circular systems do not eliminate environmental burdens but reduce and shift them toward processing stages, making energy-intensive operations such as drying and transport critical determinants of net benefits. Third, methodological difference regarding allocation choices and circular system boundaries is fundamental. From an applied perspective, the findings translate into practical recommendations for the manufactory. For brewery, the incorporation of unsold bread at moderate substitution levels (avoided barley 35%) represents an environmentally efficient and technically feasible option despite energy consumption in unsold bread stabilisation. Packaging appears as a dominant hotspot followed by boiling and hopping. However, this result is driven by the exclusion of recycling options, which leads to all packaging being modelled as virgin material. For bread-making, partial replacement of wheat flour with stabilised BSG can substantially reduce impacts, particularly those linked to crop cultivation, even when additional processing steps are required. Investments in efficient drying technologies and improved logistics for co-product collection are therefore enablers of sustainable implementation. Important contributions were additionally observed from the bakery and proofing stage. The comparison between conventional and circular configurations confirms that integrated circular systems can outperform linear ones across most EICs, with the exception of some EICs in organic productions. Overall, the fully circular scenario outperformed the Conventional System in 13 impact categories, supporting the environmental potential of circular approaches in both sectors. These results support the role of circular design as a credible pathway for improving the environmental performance of food systems. A more detailed sensitivity and uncertainty analysis could have enhanced the study’s robustness; however, it was beyond the scope of the present work which adhered to ISO standards 14040 and 14044 (2006), which do not mandate such analyses for LCA. Future research should extend this work by coupling environmental assessment with economic analyses, process scalability, and product quality and acceptability evaluations, in order to support informed decision-making by both industry and policymakers. Despite these remaining challenges, the present study provides evidence that targeted circular economy strategies can meaningfully contribute to more sustainable brewing and baking systems when grounded in life cycle thinking.

## Figures and Tables

**Table 1:** Summary of methodological assumptions distinguishing the Conventional System (CS), Environmental Charged Circular System (ECCS), and Without Environmental Charging Circular System (WECCS).

Aspect	CS—Conventional System	ECCS—Environmental Charged Circular System	WECCS—Without Environmental Charging Circular System
System boundary	Two independent cradle-to-gate systems: bread production and beer production	Integrated cradle-to-gate system linking brewing and bakery processes	Two parallel cradle-to-gate systems combined at system level
Bread production	Conventional baguette	BSG bread (20% BSG), including BSG stabilisation	BSG bread (20% BSG), including BSG stabilisation
Beer production	100% barley malt beer	Beer with 35% barley malt substitution by unsold bread	Beer with 35% barley malt substitution by unsold bread
Treatment of BSG	Not applicable	Treated as a co-product carrying environmental burdens from brewing up to the separation point	Treated as burden-free waste; only stabilisation impacts included
Treatment of unsold bread	Not applicable	Treated as a co-product carrying part of upstream bread production burdens	Treated as burden-free waste; only drying and transport included
Allocation approach	No allocation required	Environmental charging at co-product generation stage	Cut-off/burden-free approach for co-products

**Table 2:** Environmental impact categories’ values for each system and respective percentages.

*Indicator/* *System Name*	CS	ECCS	WECCS	% ECCS	% CS (Comp. with ECCS)	% CS Comp. with WECCS	% WECCS	Unit
FPMF	3 × 10^−3^	2 × 10^−3^	2 × 10^−3^	81	100	100	78	kg PM2.5 eq
FRS	3 × 10^−1^	3 × 10^−1^	3 × 10^−1^	93	100	100	87	kg oil eq
FE	3 × 10^−2^	3 × 10^−2^	3 × 10^−2^	100	99	100	97	kg 1,4–DCB
Feutr	3 × 10^−4^	3 × 10^−4^	2 × 10^−4^	79	100	100	75	kg P eq
GW	1 × 10^0^	1 × 10^0^	1 × 10^0^	84	100	100	79	kg CO_2_ eq
HCT	3 × 10^−2^	3 × 10^−2^	3 × 10^−2^	100	98	100	98	kg 1,4–DCB
HNCT	1 × 10^0^	1 × 10^0^	1 × 10^0^	97	100	100	93	kg 1,4–DCB
IR	1 × 10^0^	1 × 10^0^	1 × 10^0^	100	80	83	100	kBq Co–60 eq
LU	2 × 10^0^	1 × 10^0^	1 × 10^0^	43	100	100	38	m^2^a crop eq
ME	4 × 10^−2^	4 × 10^−2^	4 × 10^−2^	100	100	100	96	kg 1,4–DCB
Meutr	2 × 10^−3^	7 × 10^−4^	6 × 10^−4^	42	100	100	37	kg N eq
MRS	1 × 10^−2^	6 × 10^−3^	5 × 10^−3^	62	100	100	56	kg Cu eq
OF,HH	4 × 10^−3^	3 × 10^−3^	3 × 10^−3^	81	100	100	77	kg NO_x_ eq
OF,TE	4 × 10^−3^	3 × 10^−3^	3 × 10^−3^	81	100	100	78	kg NO_x_ eq
SOD	1 × 10^−5^	4 × 10^−6^	4 × 10^−6^	44	100	100	39	kg CFC11 eq
TA	1 × 10^−2^	7 × 10^−3^	6 × 10^−3^	66	100	100	63	kg SO_2_ eq
TE	5 × 10^0^	4 × 10^0^	4 × 10^0^	82	100	100	79	kg 1,4–DCB
WC	2 × 10^−2^	2 × 10^−2^	2 × 10^−2^	94	100	100	81	m^3^

CS = Conventional system; ECCS = Environmental Charged Circular System; WECCS = Without Environmental Charging Circular System.

## Data Availability

The original contributions presented in this study are included in the article. Further inquiries can be directed to the corresponding author.

## Appendix A

Additional experimental details including figures and tables are reported in Appendix A and Appendix B as cited in the article. Radar graphs for products comparison; tables reporting the inventory and the impacts and flowcharts representing system evaluation.

**Table A1:** Inventory highlights reference flow inputs and outputs for each product in order to obtain 1 L of beer (also valid for organic products). From the left: traditional beer; b.b. 35 ab (beer bread 35% avoided barley malt); b.b. 50 ab (beer bread 50% avoided barley malt).

	Unit	In/Out	Beer	b.b. 35 ab	b.b. 50 ab
**Malting**					
Electricity	kWh	In	0.140	0.140	0.140
Heat	MJ	In	2.405	2.405	2.405
Spring Barley	kg	In	1.250	1.250	1.250
Tap water	kg	In	3.000	3.000	3.000
Lorry 3.5–7.5 metric ton	kg × km	In	125.000	125.000	125.000
Barley malt	kg	Out	1.000	1.000	1.000
**Bread drying**					
Electricity	kWh	In		0.640	0.640
Sliced unsold bread	kg	In		1.000	1.000
Delivery van	kg × km	In		10.000	10.000
Dried bread	kg	Out		0.750	0.750
**Milling**					
Barley malt	kg	In	0.202	0.202	0.202
Electricity	kWh	In	0.036	0.036	0.036
Lorry 3.5–7.5 metric ton	kg × km	In	23.201	23.201	23.201
Milled malt	kg	Out	0.202	0.202	0.202
**Mashing**					
Dried unsold bread	kg	In		0.095	0.126
Electricity	kWh	In	0.002	0.002	0.002
Heat	MJ	In	0.292	0.292	0.292
Milled malt	kg	In	0.202	0.131	0.101
Tap water	kg	In	0.020	0.020	0.020
Milled malt avoided	kg	Out		0.076	0.101
Mashed wort	L	Out	0.807	0.807	0.807
**1° filtration**					
Electricity	kWh	In	0.001	0.001	0.001
Mashed wort	L	In	0.807	0.807	0.807
Tap water	kg	In	0.907	0.907	0.907
BSG	kg	Out	0.514	0.514	0.514
Wort	L	Out	1.200	1.200	1.200
**Boiling and hopping**					
Heat	MJ	In	0.403	0.403	0.403
Hop pellets	mg	In	707.000	707.000	707.000
Lorry 3.5–7.5 metric ton	kg × km	In	0.141	0.141	0.141
Wort	L	In	1.200	1.200	1.200
Boiled and hopped wort	L	Out	1.000	1.000	1.000
Water vapour	kg	Out	0.200	0.200	0.200
**Fermentation**					
Boiled and hopped wort	L	In	1.000	1.000	1.000
Electricity	kWh	In	0.006	0.006	0.006
Lorry 3.5–7.5 metric ton	kg × km	In	0.082	0.082	0.082
Yeast	kg	In	0.002	0.002	0.002
Fermented beer	L	Out	1.000	1.000	1.000
**Maturation**					
Electricity	kWh	In	0.009	0.009	0.009
Fermented beer	L	In	1.000	1.000	1.000
Matured beer	L	Out	1.000	1.000	1.000
**Cooling and bottling**					
Electricity	kWh	In	0.018	0.018	0.018
Filtered beer	L	In	1.000	1.000	1.000
Packaging glass	kg	In	0.500	0.500	0.500
Lorry 3.5–7.5 metric ton	kg × km	In	25.000	25.000	25.000
Cooled beer	L	Out	1.000	1.000	1.000

**Table A2:** Inventory highlights reference flow inputs and outputs for each bread product (also valid for organic products). From the left side: TB (traditional baguette) and BSG bread (brewery-spent grain bread).

	Unit	In/Out	TB	BSG
**Pressing**				
BSGs	kg	In		11.250
Electricity	kWh	In		0.074
Delivery van	kg × km	In		281.250
Pressed BSG	kg	Out		6.410
Water	kg	Out		4.840
**Drying**				
Electricity	kWh	In		4.170
Pressed BSG	kg	In		6.410
Dried BSG	kg	Out		2.500
Water vapour	kg	Out		3.910
**Micronisation**				
Dried BSG	kg	In		2.500
Electricity	kWh	In		0.058
Tap water	kg	In		0.830
Micronized BSG	kg	Out		2.500
Wastewater	l	Out		0.083
**Kneading**				
Yeast	g	In	6.200	6.200
Electricity	kWh	In	0.035	0.035
BSG	g			199.940
Salt	g	In	11.100	11.100
Tap water	g	In	360.600	360.600
Delivery van	kg × km	In		1.999
lorry 3.5–7.5 metric ton	kg × km	In	18.654	12.656
lorry 3.5–7.5 metric ton	kg × km	In	0.519	0.519
Wheat flour	g	In	621.800	421.860
Dough	g	Out	999.700	999.700
Wheat flour avoided	g	Out		199.940
Lorry 3.5–7.5 metric-ton avoided	kg × km	Out		5.998
**Proofing**				
Dough	g	In	999.700	999.700
Electricity	kWh	In	0.413	0.413
Leavened dough	g	Out	999.700	999.700
**Dividing**				
Electricity	kWh	In	0.003	0.003
Leavened dough	g	In	999.700	999.700
Divided dough	g	Out	999.700	999.700
**Shaping and resting**				
Divided dough	g	In	999.700	999.700
Electricity	kWh	In	0.019	0.019
Shaped loaves	g	Out	999.700	999.700
**Baking**				
Electricity	kWh	In	0.940	0.940
Shaped loaves	g	In	999.700	999.700
Baked bread	g	Out	860.700	860.700
Carbon dioxide (biogenic)	g	Out	15.995	15.995
Ethanol	g	Out	14.996	14.996
Water vapour	g	Out	108.010	108.010
**Packaging**				
baked bread	kg	In	1.000	1.000
Kraft paper	kg	In	0.100	0.100
Lorry 16–32 metric ton	kg × km	In	20.000	20.000
Packed bread traditional	kg	Out	1.000	1.000

**Table A3:** The environmental impact categories of ReCiPe 2016 Midpoint (H) and the units.

Indicator	Unit
Fine particulate matter formation	kg PM2.5 eq
Fossil resource scarcity	kg oil eq
Freshwater ecotoxicity	kg 1,4–DCB
Freshwater eutrophication	kg P eq
Global warming	kg CO_2_ eq
Human carcinogenic toxicity	kg 1,4–DCB
Human non-carcinogenic toxicity	kg 1,4–DCB
Ionizing radiation	kBq Co–60 eq
Land use	m^2^a crop eq
Marine ecotoxicity	kg 1,4–DCB
Marine eutrophication	kg N eq
Mineral resource scarcity	kg Cu eq
Ozone formation, Human health	kg NO_x_ eq
Ozone formation, Terrestrial ecosystems	kg NO_x_ eq
Stratospheric ozone depletion	kg CFC11 eq
Terrestrial acidification	kg SO_2_ eq
Terrestrial ecotoxicity	kg 1,4–DCB
Water consumption	m_3_

**Table A4:** Brewery impact categories values.

Indicator	Beer	Beer org	b.b. 35 ab	b.b. 35 AB org	b.b. 50 AB	b.b. 50 AB org	Unit
Fine particulate matter formation	1.52 × 10^−3^	1.39 × 10^−3^	1.36 × 10^−3^	1.33 × 10^−3^	1.42 × 10^−3^	1.36 × 10^−3^	kg PM2.5 eq
Fossil resource scarcity	2.20 × 10^−1^	2.13 × 10^−1^	1.98 × 10^−1^	1.96 × 10^−1^	2.07 × 10^−1^	2.03 × 10^−1^	kg oil eq
Freshwater ecotoxicity	1.30 × 10^−2^	1.19 × 10^−2^	1.18 × 10^−2^	1.15 × 10^−2^	1.26 × 10^−2^	1.20 × 10^−2^	kg 1,4–DCB
Freshwater eutrophication	1.45 × 10^−4^	1.49 × 10^−4^	1.21 × 10^−4^	1.22 × 10^−4^	1.30 × 10^−4^	1.32 × 10^−4^	kg P eq
Global warming	7.71 × 10^−1^	7.28 × 10^−1^	6.73 × 10^−1^	6.63 × 10^−1^	7.11 × 10^−1^	6.89 × 10^−1^	kg CO_2_ eq
Human carcinogenic toxicity	1.71 × 10^−2^	1.64 × 10^−2^	1.59 × 10^−2^	1.58 × 10^−2^	1.66 × 10^−2^	1.63 × 10^−2^	kg 1,4–DCB
Human non-carcinogenic toxicity	5.55 × 10^−1^	6.84 × 10^−1^	5.00 × 10^−1^	5.31 × 10^−1^	5.29 × 10^−1^	5.94 × 10^−1^	kg 1,4–DCB
Ionizing radiation	9.06 × 10^−2^	8.81 × 10^−2^	1.07 × 10^−1^	1.07 × 10^−1^	1.35 × 10^−1^	1.33 × 10^−1^	kBq Co–60 eq
Land use	4.48 × 10^−1^	7.59 × 10^−1^	1.45 × 10^−1^	2.22 × 10^−1^	2.48 × 10^−1^	4.03 × 10^−1^	m^2^a crop eq
Marine ecotoxicity	1.89 × 10^−2^	1.72 × 10^−2^	1.70 × 10^−2^	1.66 × 10^−2^	1.81 × 10^−2^	1.73 × 10^−2^	kg 1,4–DCB
Marine eutrophication	3.58 × 10^−4^	6.17 × 10^−4^	1.06 × 10^−4^	1.70 × 10^−4^	1.92 × 10^−4^	3.21 × 10^−4^	kg N eq
Mineral resource scarcity	2.94 × 10^−3^	1.48 × 10^−3^	1.77 × 10^−3^	1.41 × 10^−3^	2.21 × 10^−3^	1.48 × 10^−3^	kg Cu eq
Ozone formation, Human health	2.37 × 10^−3^	2.21 × 10^−3^	2.08 × 10^−3^	2.04 × 10^−3^	2.19 × 10^−3^	2.11 × 10^−3^	kg NO_x_ eq
Ozone formation, Terrestrial ecosystems	2.41 × 10^−3^	2.25 × 10^−3^	2.11 × 10^−3^	2.07 × 10^−3^	2.22 × 10^−3^	2.14 × 10^−3^	kg NO_x_ eq
Stratospheric ozone depletion	2.12 × 10^−6^	1.29 × 10^−6^	8.14 × 10^−7^	6.09 × 10^−7^	1.26 × 10^−6^	8.44 × 10^−7^	kg CFC11 eq
Terrestrial acidification	4.58 × 10^−3^	3.65 × 10^−3^	3.72 × 10^−3^	3.49 × 10^−3^	4.03 × 10^−3^	3.56 × 10^−3^	kg SO_2_ eq
Terrestrial ecotoxicity	2.23 × 10^0^	2.08 × 10^0^	1.94 × 10^0^	1.90 × 10^0^	2.07 × 10^0^	1.99 × 10^0^	kg 1,4–DCB
Water consumption	9.05 × 10^−3^	6.08 × 10^−3^	6.39 × 10^−3^	5.66 × 10^−3^	7.47 × 10^−3^	5.99 × 10^−3^	m_3_

**Table A5:** Bakery impact categories values.

Indicator	TB	TB org	BSG	BSG org	Unit
Fine particulate matter formation	1.09 × 10^−3^	6.07 × 10^−4^	6.64 × 10^−4^	4.91 × 10^−4^	kg PM2.5 eq
Fossil resource scarcity	1.12 × 10^−1^	1.00 × 10^−1^	9.06 × 10^−2^	8.65 × 10^−2^	kg oil eq
Freshwater ecotoxicity	1.79 × 10^−2^	1.49 × 10^−2^	1.82 × 10^−2^	1.71 × 10^−2^	kg 1,4–DCB
Freshwater eutrophication	1.87 × 10^−4^	2.19 × 10^−4^	1.29 × 10^−4^	1.40 × 10^−4^	kg P eq
Global warming	5.60 × 10^−1^	4.27 × 10^−1^	3.80 × 10^−1^	3.32 × 10^−1^	kg CO_2_ eq
Human carcinogenic toxicity	1.35 × 10^−2^	1.21 × 10^−2^	1.41 × 10^−2^	1.36 × 10^−2^	kg 1,4–DCB
Human non-carcinogenic toxicity	6.01 × 10^−1^	5.33 × 10^−1^	5.76 × 10^−1^	5.52 × 10^−1^	kg 1,4–DCB
Ionizing radiation	1.06 × 10^0^	1.06 × 10^0^	1.27 × 10^0^	1.27 × 10^0^	kBq Co–60 eq
Land use	1.44 × 10^0^	2.75 × 10^0^	5.71 × 10^−1^	1.04 × 10^0^	m^2^a crop eq
Marine ecotoxicity	2.35 × 10^−2^	1.99 × 10^−2^	2.37 × 10^−2^	2.24 × 10^−2^	kg 1,4–DCB
Marine eutrophication	1.40 × 10^−3^	2.43 × 10^−3^	5.40 × 10^−4^	9.08 × 10^−4^	kg N eq
Mineral resource scarcity	6.87 × 10^−3^	1.85 × 10^−3^	3.77 × 10^−3^	1.98 × 10^−3^	kg Cu eq
Ozone formation, Human health	1.79 × 10^−3^	1.39 × 10^−3^	1.14 × 10^−3^	1.00 × 10^−3^	kg NO_x_ eq
Ozone formation, Terrestrial ecosystems	1.81 × 10^−3^	1.42 × 10^−3^	1.17 × 10^−3^	1.03 × 10^−3^	kg NO_x_ eq
Stratospheric ozone depletion	7.68 × 10^−6^	3.87 × 10^−6^	3.02 × 10^−6^	1.66 × 10^−6^	kg CFC11 eq
Terrestrial acidification	5.44 × 10^−3^	1.71 × 10^−3^	2.59 × 10^−3^	1.26 × 10^−3^	kg SO_2_ eq
Terrestrial ecotoxicity	2.47 × 10^0^	2.26 × 10^0^	1.78 × 10^0^	1.70 × 10^0^	kg 1,4–DCB
Water consumption	1.24 × 10^−2^	8.25 × 10^−3^	1.09 × 10^−2^	9.42 × 10^−3^	m_3_
